# Supplementary material for: Dietary Profile and Nutritional Status of the Roma Population Living in Segregated Colonies in Northeast Hungary
Source: Nutrients. 2020 Sep 16;12(9):2836. doi: 10.3390/nu12092836 (PMC7551568; doi:10.3390/nu12092836)
Supplement: Supplementary file 1 [file nutrients-12-02836-s001.pdf]

Dietary data

Design, piloting and validation of dietary data collection instrument

<u>Dietary intake data collection</u>

Dietary intake data were obtained through an interviewer-assisted, double multiple-pass 24-h diet recall (one weekday and one weekend day). Prior to the cross-sectional survey, two focus groups were organized with Hungarian general (HG) and Hungarian Roma (HR) subjects in two different locations (Nyíregyháza and Debrecen). A single 24-hour recall was completed, in order to pilot and compare the 24-hour multiple-pass method (MPM) recall instrument against three-day unweighted food record. Questions concerning comprehension and interpretation of the illustrated accompanying draft food booklet, recognition and familiarity with different dishes, food items and containers, as well as potential omittable or under-reportable items were also examined. Images of canned foods commonly consumed, usual utensils and some containers widely used among subjects, not present in the initial draft booklet, were inserted in the latter version accordingly.

<u>Booklet development</u>

Following focus groups' findings and the results from the recall instrument, modifications were introduced to the accompanying booklet, such as representations of real-life size drawings of different containers and dishes. This was done with the intent of making it more convenient for participants to identify and estimate amounts and portion sizes of reported food and drinks, as well as to increase accuracy and ease of identification of the illustrated items. Since, to date there is no standard portion size guide booklet for Hungary, further adjustments were made. A recognizable life-size picture of a 200 HUF (Hungarian Forints) coin and occasionally other recognizable items (kitchen utensils) were attached to picture models to increase accuracy of estimating portion sizes (*see example in Figure S1*).

Figure S1. Sample quantification from the booklet used in this study

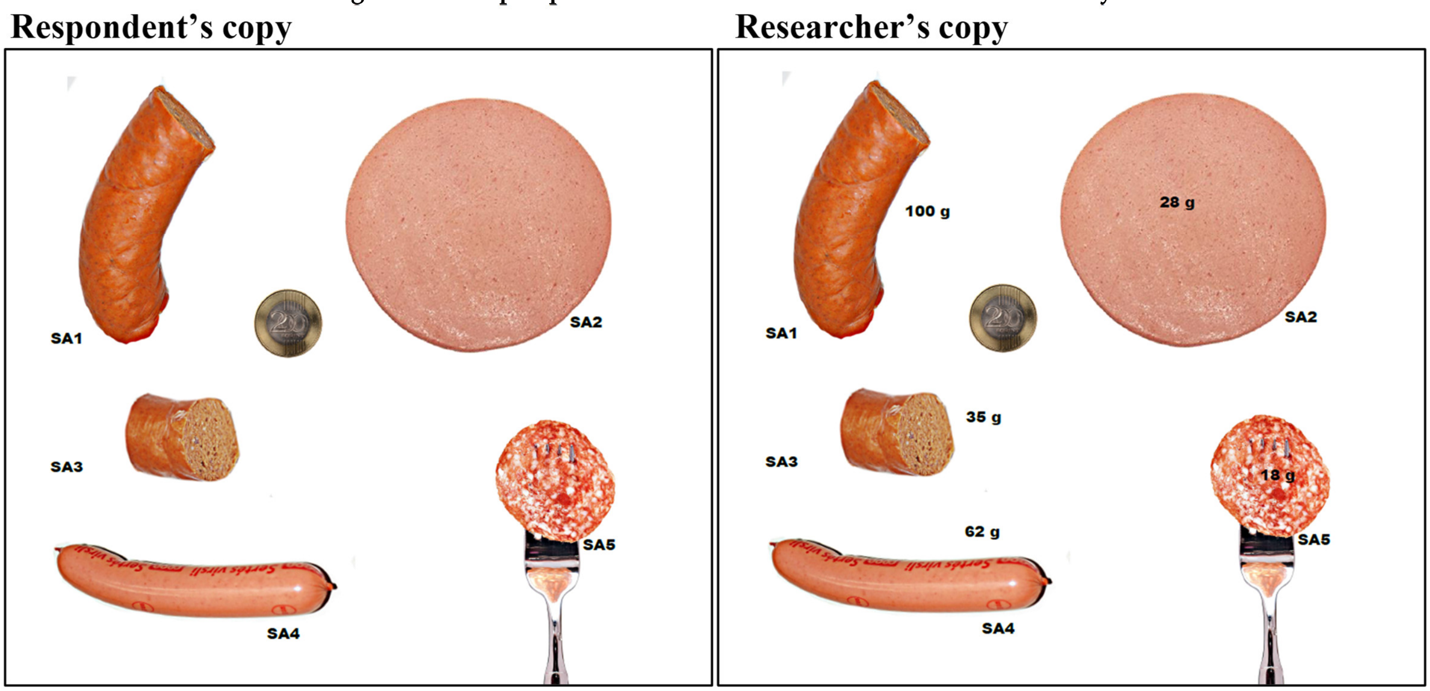

Portion sizes for fruits, vegetables and other food/drink items were established and a database with food measurements was developed with average weights of different items after sampling and weighting with a digital kitchen scale. In addition, two versions of the booklet were developed, one for the interviewers and one for participants. The purpose for creating two versions was to minimize influence on participants during portion size estimation. The interviewer's version had clear indications of exact weight of food/drink(s), but participant's version had no indication of any quantity or weight (see example in *Figure S1* above).

The booklet was validated qualitatively through focus-groups and piloting before beginning the study as described above and the design process was developed from the concept used in our previous research [1].

Dietary data processing

Following data collection completion, a quality appraisal protocol was applied and completed recalls would be accepted if: (1) two recalls were available (i.e. one for a weekday and one for a weekend day), (2) subject identification code was correct, (3) columns were filled according to instructions. In case of substantial error(s), interviewers should have repeated the recall procedure for the corresponding subject. After successful initial assessment, recalls were considered eligible for data entry.

Thirty-five undergraduate students of the dietetics bachelor program, at the Faculty of Public Health, University of Debrecen, were recruited and trained on the process of data entry in *NutriComp Étrend* software. The process was supervised by a registered dietitian. Data entry included three stages: firstly, data were entered in the database, based on the dietary recalls and all missing items or unusual foods/drinks were indicated. Secondly, the supervisor determined which relevant foods, drinks, ingredients or recipes could be used in these cases, and eventually, the correct information was inserted and processed.

Table S1. Socio-demographic characteristics of excluded subjects due to implausibility of intake data

**Variable**		Hungarian General (51)	Hungarian Roma (43)
**Age – mean (std. deviation)**		45 (13)	42 (12)
**Sex**	*Male*	9(64.3)	5(35.7)
	Female	42(52.5)	38(47.5)
**Perceived financial status**	*Very good*	2(100)	0(0)
	Good	10(58.8)	7(41.2)
	Fair	37(63.8)	21(36.2)
	Challenging	1(11.1)	8(88.9)
	Very challenging	1(14.3)	6(85.7)
**Educational level**	*Elementary*	10(22.2)	35(77.8)
	Secondary	17(89.5)	2(10.5)
	Vocational training	11(68.8)	5(31.3)
	University degree	13(92.9)	1(7.1)
**Economic activity**	*Full-time employment*	35(53.8)	30(46.2)
	Part-time employment	5(83.3)	1(16.7)
	Student	1(100)	0(0)
	Retired	3(50)	3(50)
	Ill-health retirement	3(60)	2(40)
	Unemployed	4(36.4)	7(63.6)

Values are presented as n (%), unless otherwise indicated.
Only data for participants with full records are presented.

Table S2. Socio-demographic characteristics of excluded subjects due to implausibility of intake data, by type of implausibility (i.e. low vs. high)

		Hungarian General			Hungarian Roma		
**Intake**		*Implausibly Low*	*Implausibly High*	**Included**	*Implausibly Low*	*Implausibly High*	**Included**
**Age – mean (std. deviation)**		51(11)	45(13)	44.2 (12.2)	52(12)	41(12)	42.9 (12.1)
Females		2(5)	38(95)	188 (52.4)	3(7.9)	35(92.1)	248 (72.1)
**Perceived financial status**	*Very good*	0(0)	2(100)	18 (5.2)	0(0)	0(0)	5 (1.5)
	Good	0(0)	10(100)	97 (27.6)	1(14.3)	6(85.7)	46 (13.5)
	Fair	4(11.1)	32(88.9)	190 (54.1)	1(4.8)	20(95.2)	186 (54.7)
	Challenging	0(0)	1(100)	40 (11.4)	0(0)	8(100)	85 (25.0)
	Very challenging	0(0)	0(0)	6 (1.7)	2(33.3)	4(66.7)	18 (5.3)
**Educational level**	*Elementary*	0(0)	9(100)	76 (21.2)	4(11.4)	31(88.6)	292 (84.9)
	Secondary	1(6.3)	15(93.8)	118 (32.9)	0(0)	2(100)	17 (4.9)
	Vocational training	3(27.3)	8(72.7)	112 (31.2)	0(0)	5(100)	35 (10.2)
	University degree	0(0)	13(100)	53 (14.7)	0(0)	1(100)	0 (0.0)
**Economic activity**	*Full-time employment*	3(8.6)	32(91.4)	267 (74.4)	3(10)	27(90)	233 (67.7)
	Part-time employment	1(25)	3(75)	29 (8.1)	0(0)	1(100)	23 (6.7)
	Student	0(0)	1(100)	8 (2.2)	0(0)	0(0)	0 (0.0)
	Retired	0(0)	3(100)	22 (6.1)	0(0)	3(100)	22 (6.4)
	Ill-health retirement	0(0)	3(100)	18 (5.0)	0(0)	2(100)	10 (2.9)
	Unemployed	0(0)	3(100)	15 (4.2)	1(14.3)	6(85.7)	56 (16.3)

Values are presented as n (%), unless otherwise indicated. Included intakes are highlighted in grey for reader's convenience and only data for participants with full records are presented.

Table S3. Macronutrient intakes among Hungarian Roma and general populations, by sex

**Macronutrient**	**Hungarian General (n=359)**		**Hungarian Roma (n=344)**		**Recommendation [*Ref.*]**
	**Males**	**Females**	**Males**	**Females**	
Carbohydrates (%E)	46.4(45;47.7)	46.1(44.8;47.4)	48.8(47;50.6)	48(46.8;49.1)	**55-75 %E [2]**
Sugar (*g*)	98(87.2;108.8)	94.7(84.9;104.5)	113.1(96.9;129.3)	96.9(88.9;104.9)	**≤ 31 g [3]**
Sugar (*%E*)	16.8(15.3;18.2)	17.3(15.9;18.7)	19.8(17.7;21.9)	18.4(17.2;19.6)	**≤10 %E (≤5%E) [2]**
Fiber (*g*)	20.8(19.3;22.3)	19.9(18.6;21.3)	19.1(17.5;20.6)	20.9(19.2;22.6)	**≥ 24 g [2]; ≥ 42.9 g[3]**
Fiber (*g/1000 kcal*)	9.5(8.9;10.1)	9.8(9.2;10.4)	8.9(8.3;9.5)	10.3(9.7;11)	**14.8 g/1000 kcal [4]**
Proteins (%E)	15.3(14.9;15.8)	15.9(15.3;16.4)	14.9(14.2;15.6)	15.1(14.7;15.5)	**10-15 %E [2]**
Animal-based proteins (*% tot. proteins*)	83.2(82.2;84.3)	82.6(81.5;83.8)	82.1(80.7;83.4)	81.7(80.7;82.6)	-
Plant-based protein (*% tot. proteins*)	16.8(15.7;17.8)	17.4(16.2;18.5)	17.9(16.6;19.3)	18.3(17.4;19.3)	-
Animal/plant protein ratio	0.77(0.68;0.86)	0.73(0.67;0.79)	0.78(0.71;0.86)	0.86(0.78;0.94)	
Amino acids (*g*)	78.3(74.1;82.5)	75.4(71.4;79.4)	72.7(68.1;77.2)	70.5(67.4;73.7)	-
Essential amino acids (*g*)	29.3(27.7;30.9)	28.2(26.7;29.7)	26.8(25.1;28.6)	26.2(25;27.4)	-
Fats	37.5(36.2;38.8)	36.9(35.7;38.1)	35.8(34;37.5)	36.3(35.2;37.3)	**15-30 %E [2]**
Animal-based fats (*% of total fats*)	60.3(57.7;62.9)	58.3(55.9;60.6)	58.3(54.8;61.8)	61.5(59.5;63.6)	-
Plant-based fats (*% of total fats*)	39.7(37.1;42.3)	41.7(39.4;44.1)	41.7(38.2;45.2)	38.5(36.4;40.5)	-
SFA (*%E*)	11.1(10.5;11.7)	10.3(9.8;10.8)	10.3(9.6;11)	10.8(10.4;11.2)	**≤10 %E [2]**
MUFA (*%E*)	12.2(11.5;12.8)	11.7(11.1;12.2)	11.2(10.4;12)	11.5(11.1;12)	-
PUFA (*%E*)	8.9(8.5;9.4)	9.1(8.7;9.5)	8.5(8.0;9.0)	8.1(7.7;8.5)	**6-10 %E [2]**
UFA (*%E*)	21(20.2;21.8)	20.7(20;21.5)	19.7(18.6;20.7)	19.6(19.0;20.3)	-
Cholesterol (*mg/1000 kcal*)	166(154.6;177.5)	179.2(167.5;190.8)	159.2(147;171.5)	159.6(150.6;168.6)	**71.4 mg/1000 kcal [4]**
Cholesterol (*mg*)	368(340.4;395.6)	370.3(345.3;395.4)	344.1(314.2;374.1)	338.1(313.6;362.6)	**<300 mg[2]; ≤ 125.2[3]**
ω-3 fatty acids (*%E*)	0.32(0.27;0.37)	0.31(0.28;0.34)	0.27(0.24;0.31)	0.27(0.25;0.29)	**1-2 % [2]**
ω-6 fatty acids (*%E*)	8.6(8.1;9)	8.8(8.4;9.2)	8.2(7.7;8.7)	7.9(7.5;8.2)	**5-8 % [2]**
α-linolenic acid (*%E*)	0.26(0.25;0.28)	0.28(0.26;0.3)	0.25(0.24;0.27)	0.25(0.24;0.27)	**0.5-2 %E [5]**

Notes: Every value is given as mean and 95% confidence interval of the mean. 95% CI: 95% confidence interval of the mean; [Ref.]: Reference – source of the recommended range; MUFAs: monounsaturated fatty acids; PUFA: polyunsaturated fatty acids; SFA: saturated fatty acids; UFA: unsaturated fatty acids; (%E): intake as percentage of total energy.

Table S4. Micronutrient intakes among Hungarian Roma and general populations, by sex

**Micronutrients**	**Recommendation [Ref.]**	**Hungarian General (n=359)**		**Hungarian Roma (n=344)**	
Minerals and trace elements		*Males*	*Females*	*Males*	*Females*
Magnesium (*mg/1000 kcal*)	**≥238 mg/ 1000 kcal [4]**	199.84(143.77;255.92)	186.98(176.76;197.21)	174.45(161.27;187.64)	184.42(174.72;194.11)
Calcium (*mg/1000 kcal*)	**≥590 mg/ 1000 kcal [4]**	237.75(219.83;255.66)	258.29(234.68;281.89)	253.64(229.74;277.54)	245.87(229.32;262.42)
Sodium (*mg/1000 kcal*)	**≤1143 mg/1000 kcal [4]**	2698.64(2509.95;2887.33)	2563.98(2458.06;2669.91)	2257.98(2130.44;2385.51)	2535.81(2418.26;2653.37)
Sodium (*mg*)	**≤2000 mg [6]**	5991.42(5503.17;6479.67)	5327.94(4992.57;5663.3)	4850.5(4505.17;5195.83)	5188.77(4901.07;5476.47)
Potassium (*mg/1000 kcal*)	**≥2238 mg/ 1000kcal [4]**	1376.57(1242.84;1510.3)	1404.31(1311.62;1497.01)	1384.59(1226.4;1542.78)	1454.98(1358.45;1551.52)
Potassium (*mg*)	**≥3510 mg [7]**	3120.91(2688.64;3553.17)	2855.31(2658.11;3052.51)	3001.36(2596.01;3406.72)	2960.12(2740.58;3179.66)
Iron (*mg/1000 kcal*)	-	5.05(4.77;5.34)	5.41(4.99;5.83)	4.68(4.37;4.99)	5.48(5.06;5.91)
Iron (*mg*)	**1.05 mg [8]**	11.28(10.4;12.15)	11.11(10.2;12.02)	10.09(9.25;10.93)	11.43(10.34;12.53)
Vitamins					
Vitamin A (*μg/1000 kcal*)	-	135.67(114.96;156.38)	142.65(118.27;167.03)	138.48(88.63;188.32)	183.19(129;237.38)
Vitamin A (*μg RE*)	**500 μg RE [8]**	0.5(0.44;0.55)	0.51(0.45;0.58)	0.50(0.41;0.59)	0.68(0.51;0.84)
Beta-carotene (*mg/1000 kcal*)	-	1.06(0.91;1.2)	1.33(1.12;1.55)	1.18(0.92;1.44)	1.42(1.21;1.64)
Vitamin B_1 (*μg/1000 kcal*)	-	467.22(442.94;491.51)	482.9(457.5;508.3)	427.69(394.82;460.55)	474.82(452.4;497.24)
Vitamin B_1 (*μg*)	**≥1100 μg [8]**	1048.02(971.2;1124.83)	1000.46(935.3;1065.62)	924.18(838.38;1009.97)	974.6(916.23;1032.97)
Vitamin B_2 (*μg/1000 kcal*)	-	595.6(470.29;720.92)	556.5(523.49;589.51)	516.45(466.77;566.12)	548.61(520.23;576.99)
Vitamin B_2 (*μg*)	**≥1100 μg [8]**	1452.16(936.36;1967.96)	1143.06(1060.79;1225.33)	1124.21(999.28;1249.13)	1139.74(1059.33;1220.15)
Vitamin B_6 (*μg/1000 kcal*)	-	812.19(771.75;852.63)	851.07(809.34;892.8)	743.55(686.72;800.38)	781.71(746.21;817.22)
Vitamin B_6 (*μg*)	**≥1300 μg [8]**	1790.47(1679.05;1901.89)	1735.48(1639.76;1831.19)	1586.25(1452.74;1719.76)	1593.88(1505.68;1682.08)
Vitamin B_{12} (*μg/1000 kcal*)	-	2.25(0.14;4.37)	1.2(1.04;1.35)	1.08(0.79;1.36)	1.49(0.78;2.21)
Vitamin B_{12} (*μg*)	**≥2.4 μg [8]**	5.18(0.26;10.11)	2.47(2.14;2.8)	2.36(1.62;3.1)	3.22(1.66;4.77)
Vitamin B_3 (*mg NE/1000 kcal*)	**≥6.6 mg NE/1000 kcal [9]**	10.65(6.91;14.38)	9.39(8.69;10.08)	7.84(7.07;8.6)	8.75(8.25;9.26)
Vitamin B_3 (*mg NE*)	**≥14 mg NE [8]**	44.41(29.2;59.62)	35.36(33.41;37.31)	32.56(30.09;35.04)	33.32(31.55;35.09)
Vitamin C (*mg/1000 kcal*)	-	35.66(30.58;40.74)	40.26(34.87;45.65)	36.55(27.51;45.59)	41.38(36.43;46.33)
Vitamin C (*mg*)	**≥45 mg [8]**	76.61(66.49;86.73)	80.88(70.64;91.12)	76.41(58.7;94.13)	80.52(71.56;89.48)
Vitamin D (*mg/1000 kcal*)	-	0.81(0.68;0.95)	0.77(0.68;0.85)	0.77(0.68;0.86)	0.79(0.6;0.97)
Vitamin D (*μg*)	**≥10 μg [8]**	1.83(1.51;2.15)	1.61(1.43;1.79)	1.69(1.46;1.92)	1.68(1.26;2.09)

References

1. Llanaj E, Ádány R, Lachat C, D'Haese M. Examining food intake and eating out of home patterns among university students. Plos ONE. 2018;13(10):e0197874. doi: 10.1371/journal.pone.0197874.
2. Nishida C, Uauy R, Kumanyika S, Shetty P. The joint WHO/FAO expert consultation on diet, nutrition and the prevention of chronic diseases: process, product and policy implications. Public Health Nutr. 2004;7(1a):245-50. doi: 10.1079/phn2003592
3. Willett W, Rockström J, Loken B, Springmann M, Lang T, Vermeulen S, et al. Food in the Anthropocene: the EAT–Lancet Commission on healthy diets from sustainable food systems. Lancet. 2019;393(10170):447-92. doi: 10.1016/S0140-6736(18)31788-4.
4. Mellen PB, Gao SK, Vitolins MZ, Goff DC, Jr. Deteriorating Dietary Habits Among Adults With Hypertension: DASH Dietary Accordance, NHANES 1988-1994 and 1999-2004. JAMA Intern Med. 2008;168(3):308-14. doi: 10.1001/archinternmed.2007.119.
5. Elmadfa I, Kornsteiner M. Fats and Fatty Acid Requirements for Adults. Ann Nutr Metab. 2009;55(1-3):56-75. doi: 10.1159/000228996.
6. World Health Organization. Guideline: sodium intake for adults and children Geneva, Switzerland 2012 [29.06.2020]. Available from: https://www.who.int/nutrition/publications/guidelines/sodium_intake_printversion.pdf.
7. World Health Organization. Guideline: potassium intake for adults and children Geneva, Switzerland 2012 [29.06.2020]. Available from: https://apps.who.int/iris/bitstream/handle/10665/77986/9789241504829_eng.pdf?sequence=1.
8. WHO/FAO. Vitamin and mineral requirements in human nutrition: World Heath Organization and Food and Agriculture Organization; 2005 [29.06.2020]. Available from: https://apps.who.int/iris/bitstream/handle/10665/42716/9241546123.pdf.
9. EFSA. Dietary Reference Values for nutrients Summary report. EFSA Supporting Publications. 2017;14(12):e15121E. doi: 10.2903/sp.efsa.2017.e15121.
